# Prognostic Stratification Based on HIF-1 Signaling for Evaluating Hypoxic Status and Immune Infiltration in Pancreatic Ductal Adenocarcinomas

**DOI:** 10.3389/fimmu.2021.790661

**Published:** 2021-12-03

**Authors:** Hongkai Zhuang, Shujie Wang, Bo Chen, Zedan Zhang, Zuyi Ma, Zhenchong Li, Chunsheng Liu, Zixuan Zhou, Yuanfeng Gong, Shanzhou Huang, Baohua Hou, Yajin Chen, Chuanzhao Zhang

**Affiliations:** ^1^Department of General Surgery, Guangdong Provincial People’s Hospital, Guangdong Academy of Medical Sciences, Guangzhou, China; ^2^Department of Hepatobiliary Surgery, Sun Yat-Sen Memorial Hospital, Sun Yat-Sen University, Guangzhou, China; ^3^Department of Breast Cancer, Cancer Center, Guangdong Provincial People’s Hospital, Guangdong Academy of Medical Sciences, Guangzhou, China; ^4^Department of Urology, Peking University First Hospital, Beijing, China

**Keywords:** PDAC, HIF-1, hypoxia, ICB, immunosuppression, immune infiltration

## Abstract

Pancreatic ductal adenocarcinoma (PDAC) has a hypoxic and desmoplastic tumor microenvironment (TME), leading to treatment failure. We aimed to develop a prognostic classifier to evaluate hypoxia status and hypoxia-related molecular characteristics of PDAC. In this study, we classified PDAC into three clusters based on 16 known hypoxia-inducible factor 1 (HIF-1)-related genes. Nine differentially expressed genes were identified to construct an HIF-1 score system, whose predictive efficacy was evaluated. Furthermore, we investigated oncogenic pathways and immune-cell infiltration status of PDAC with different scores. The C-index of the HIF-1score system for OS prediction in the meta-PDAC cohort and the other two validation cohorts were 0.67, 0.63, and 0.65, respectively, indicating that it had a good predictive value for patient survival. Furthermore, the area under the curve (AUC) of the receiver operating characteristic (ROC) curve of the HIF-1α score system for predicting 1-, 3-, and 4-year OS indicated the HIF-1α score system had an optimal discrimination of prognostic prediction for PDAC. Importantly, our model showed superior predictive ability compared to previous hypoxia signatures. We also classified PDAC into HIF-1 scores of low, medium, and high groups. Then, we found high enrichment of glycolysis, mTORC1 signaling, and MYC signaling in the HIF-1 score high group, whereas the cGMP metabolic process was activated in the low score group. Of note, analysis of public datasets and our own dataset showed a high HIF-1 score was associated with high immunosuppressive TME, evidenced by fewer infiltrated CD8^+^ T cells, B cells, and type 1 T-helper cells and reduced cytolytic activity of CD8^+^ T cells. In summary, we established a specific HIF-1 score system to discriminate PDAC with various hypoxia statuses and immune microenvironments. For highly hypoxic and immunosuppressive tumors, a combination treatment strategy should be considered in the future.

## Introduction

Pancreatic ductal adenocarcinoma (PDAC) is one of the deadliest malignancies and accounts for nearly 4.5% of all cancer-related deaths worldwide, with a 5-year survival rate of less than 10% (1, 2). Despite major efforts to improve the diagnosis and treatment of PDAC, the survival rate of patients with PDAC has not significantly improved (3). In particular, novel treatments were found to have limited indications or low response rates (2–5). For example, olaparib, is only effective in patients with germline BRCA mutations (6–8). PD-1/PD-L1 inhibition-based immunotherapy is under investigation, and preliminary data showed limited efficacy for single drug treatment (9, 10). These are due to tumor heterogeneity and the specific tumor microenvironment (TME) in PDAC (11–13). In addition, the traditional prognostic clinicopathological characteristics, such as American Joint Committee on Cancer (AJCC) stage and histologic grade, have less accurate predictive value for the clinical outcome of patients with PDAC (14–16). Therefore, exploring the molecular classification and mechanisms leading to TME development and tumor progression will help in designing more effective precision treatments for PDAC.

Desmoplasia and hypoxia are the major characteristics of TME in PDAC, in which desmoplasia worsens tumor hypoxia, and hypoxic conditions promote the proliferation of stromal cells such as CAFs, leading to severe desmoplasia (17–19). Hypoxia-inducible factor-1 (HIF-1) is a master regulator of tumor hypoxia and plays a critical role in promoting the malignant phenotypes of PDAC (20, 21). For example, HIF-1 was reported to enhance the transcription of Snail by binding to its hypoxia response elements, inducing epithelial-mesenchymal transition and cancer metastasis in PDAC (22). In addition, the hypoxic TME of PDAC could upregulate the expression of multidrug resistance 1 (MDR1) through the HIF-1 signaling pathway, thereby mediating chemotherapy resistance (23). Importantly, HIF-1 regulates anti-tumor immunity by regulating the expression of PD-L1 or CD47, resulting in an immunosuppressive TME (24–27). Thus, hypoxia and HIF-1 may affect the expression of different genes and lead to corresponding cancer cell behaviors. Therefore, it is of great interest to establish a prognostic classifier to evaluate the different hypoxia status and characteristics of hypoxia-related subtypes of PDAC.

In the current study, using multiple bioinformatics analysis, we classified patients with PDAC into three clusters based on HIF-1 related genes. Nine differentially expressed genes among the three HIF-1 clusters were identified from the meta-PDAC cohort to construct an HIF-1 score system for prognostic stratification of patients with PDAC. The predictive efficacy of the HIF-1 score system was evaluated in meta-PDAC cohort and validation cohort. Of note, we also comprehensively assessed the oncologic biological pathways and immune-cell infiltration status for pancreatic cancers with different HIF-1 scores.

## Materials And Methods

### PDAC Datasets and Preprocessing

Publicly available PDAC mRNA-sequencing data and corresponding clinical information of patients were obtained from the Gene Expression Omnibus (GEO, https://www.ncbi.nlm.nih.gov/geo/), the Cancer Genome Atlas (TCGA, https://cancergenome.nih.gov/), and the International Cancer Genome Consortium (ICGC, https://icgc.org/) databases. The PDAC dataset GSE62452 (Platform: GPL6244; 61 non-tumor samples and 69 tumor samples) from the GEO database and TCGA PDAC dataset (4 non-tumor samples and 146 tumor samples) were integrated into a meta-PDAC cohort (65 non-tumor samples and 215 tumor samples). The R package ‘sva’ was used to eliminate batch effects. Of the 215 PDAC cases in the meta-PDAC cohort, 205 were cases with OS > 1 month and were used as the training cohort for prognostic stratification based on HIF-1 signaling. Transcriptomic data from the ICGC PDAC cohort (N = 96; validation cohort 1) and GSE79668 cohorts (Platform: GPL11154; N = 51; validation cohort 2) were used for validation.

### Identification of HIF-1 Related Genes in PDAC

A total of 16 HIF-1 related genes were identified, corroborating previous studies (28, 29). The differential expression of these genes between non-tumor and tumor samples was evaluated in the meta-PDAC cohort. The prognostic value of HIF-1-related genes in PDAC was evaluated in a meta-PDAC cohort using Kaplan–Meier (KM) survival analysis.

### Consensus Clustering Analysis

Using the R package ‘ConsensusClusterPlus’, consensus clustering was conducted to categorize PDAC patients into subgroups based on the expression of HIF-1 related genes. Principal component analysis (PCA) was performed to evaluate the clustering efficacy. KM survival analysis was then performed to assess the OS difference between different subgroups. The differential expression of HIF-1 related genes between different subgroups was visualized using the R package ‘pheatmap’. Then, the association between subgroups and clinicopathological characteristics, including AJCC stage and histologic grade, was evaluated using the chi-square test or Fisher’s exact test. Differentially expressed genes between different subgroups were identified using the R package ‘limma’ under the threshold of |log2 fold change (FC)| > 0.5. The overlapping differentially expressed genes (ODEGs) were selected for subsequent analysis.

### Development and Validation of the HIF-1 Score System

Using the R package ‘survival’, we performed univariate Cox regression analysis to assess the association between the ODEGs and OS in batches in the meta-PDAC cohort. Then, using the R package ‘glmnet’, the critical prognosis-associated ODEGs were further determined through least absolute shrinkage and selection operator (LASSO) regression analysis. KM survival analysis was also conducted to evaluate the prognostic association of the critical prognosis-associated ODEGs according to the optimal cutoff point using the R package ‘survminer’. Using the R package ‘survival’, an HIF-1 score system was developed based on a linear combination of the expression of the critical prognosis-associated ODEGs and the multivariable Cox regression coefficients as the weight. For external validation of the HIF-1 score system, the HIF-1 score was also calculated for patients with PDAC in the ICGC PDAC cohort and GSE79668 cohort with the same multivariable Cox regression coefficients in the meta-PDAC cohort.

Using the R package ‘survminer’, KM survival curves for OS were constructed according to the optimal cutoff points obtained from X-tile software version 3.6.1 (low HIF-1 score, medium HIF-1 score, and high HIF-1 score) (30). The predictive performance of the HIF-1 score system was evaluated using the C-index and AUC of the ROC curves. PCA was performed to evaluate prognostic stratification efficacy. In addition, the association between HIF-1 score and clinicopathological characteristics, including AJCC stage and histologic grade, was evaluated using the chi-square test.

### Association Between HIF-1 Score and Somatic Mutation in PDAC

Using the R package ‘maftools’, we visualized the somatic mutation profile of PDAC in the TCGA PDAC cohort. We further investigated the association between HIF-1 score and somatic mutation using the chi-square test. We also assessed the association between tumor mutation burden (TMB) and HIF-1 score. In summary, we aimed at preliminarily determining whether somatic mutation status affected hypoxia status in PDAC.

### Gene Set Variation Analysis (GSVA)

GSVA is an analytical method used to calculate the enrichment scores of specific gene sets for each sample based on RNA-seq (31). Using the R package ‘gsva’, we conducted GSVA to estimate the enrichment scores of 50 gene ontology gene sets (h.all.v7.0.symbols.gmt), and 231 metabolic process gene sets (c5.go.bp.v7.4.symbols.gmt) obtained from the Molecular Signature Database (MSigDB, http://software.broadinstitute.org/gsea/msigdb) in the meta-PDAC cohort. Furthermore, GSVA was also implanted to calculate the enrichment scores of 25 immune-related terms extracted from previous studies (32).

### Association Between HIF-1 Score and Hypoxia Scores

A total of three hypoxia scores based on the TCGA PDAC dataset were obtained from the cBioportal database (http://www.cbioportal.org/), including Buffa hypoxia, Ragnum hypoxia score, and Winter hypoxia score (33–35). We then evaluated the differences in these three hypoxia scores between different HIF-1 score subgroups. Correlation analysis between the HIF-1 score and these three hypoxia scores was conducted using Pearson correlation coefficients. KM survival curves were obtained for HIF-1 and the three hypoxia scores in the sample TCGA PDAC cohort. Then, ROC curves for 1-, 2-, and 3-year OS were performed for HIF-1 score and these three hypoxia scores to compare their predictive reliability for OS.

### Real-Time Polymerase Chain Reaction (RT-qPCR)

A total of 28 samples of PDAC patients were collected after surgical resection in Guangdong Provincial People’s Hospital during 2015-2021. The total mRNA of PDAC tissues were isolated using TRIzol (Invitrogen, USA) following the manufacturer’s instruction. The RT-qPCR was conducted in triplicate using Taqman™ Assay kit (Applied Biosystems, USA). The expressions were estimated by 2^-△△Ct^ method andβ-actin was used as an internal control. The sequences of primers for the nine genes of our model were shown in **Table S1**. Informed consent was obtained from all patients, and the study was approved by the Ethics Committee of Guangdong Provincial People’s Hospital.

### Immunohistochemistry

The formalin-fixed paraffin-embedded sections (4-µm thick) from the corresponding PDAC samples were deparaffinized and rehydrated, followed by antigen retrieval using citrate buffer (pH 8.0). Staining for CD8+ T cells were performed using a rabbit anti-CD8A monoclonal antibody (GB13429, Servicebio, China) and the LSAB+ System HRP kit (DAKO, Carpineteria, CA) according to the manufacturer’s instructions. The levels of CD8A-positive cells was quantified by whole slide digital scanning using Aperio VERSA scanner (Leica Bioosystems, USA), and converted to number/mm^2^.

### Statistical Analysis

GraphPad Prism 8.0 software (GraphPad Software, Inc.) and the R software version 3.5.2 (http://r-project.org/) were used to conduct all statistical analyses. Group differences analysis were performed using Wilcoxon test or Kruskal–Wallis test, and expressed as means ± standard deviation (SD). Pearson correlation coefficient was used for correlation analysis. A two-sided P value < 0.05 was considered as statistically significant.

## Results

### The mRNA Expression of HIF-1 Related Genes in PDAC

We analyzed 16 known HIF-1 related genes and found that 15 were significantly overexpressed in tumor samples in the meta-PDAC cohort, including *ALDOA*, *ALDOC*, *ENO1*, *GAPDH*, *HIF1A*, *HK1*, *HK2*, *LDHA*, *PDK1*, *PFKFB3*, *PFKL*, *PGK1*, *PKM*, *SLC2A1*, and *SLCS2A3*, whereas only one (*BNIP3*) was significantly downregulated (**Figure S1A**). KM survival analysis demonstrated that 13 HIF-1 related genes were significantly associated with shorter OS of PDAC patients, namely *ALDOA*, *ALDOC*, *ENO1*, *GAPDH*, *HIF1A*, *HK1*, *HK2*, *LDHA*, *PDK1*, *PGK1*, *PKM*, *SLC2A1*, and *SLCS2A3*. (**Figure S1B**).

### Consensus Clustering Analysis Identified Three HIF-1 Clusters of PDAC With Different Clinical Outcomes

**Figure 1:**
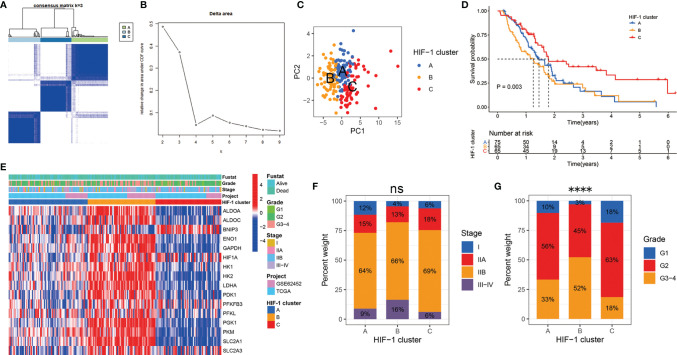
Consensus clustering analysis identified three HIF-1 clusters of PDAC with different clinical outcomes. **(A)** The meta-PDAC cohort was divided into three distinct clusters when k = 3. **(B)** Relative change in area under consensus clustering cumulative distribution function curve for k = 2 to 9. **(C)** PCA demonstrated that HIF-1 related genes worked well with significant clustering efficacy. **(D)** The KM survival curve for OS showed a significant difference among these three HIF-1 clusters. **(E)** Heatmap to show the 16 HIF-1 related genes expression and corresponding clinicopathological information in the three HIF-1 clusters. **(F)** HIF-1 cluster was not significantly associated with AJCC stage. **(G)** HIF-1 cluster was significantly associated with histologic grade. PDAC, pancreatic ductal adenocarcinoma; PCA, principal component analysis; KM, Kaplan-Meier; OS, overall survival; AJCC, American Joint Committee on Cancer. ns > 0.05; ****P value < 0.0001.

According to the mRNA expression similarity of HIF-1 related genes, k = 3 was the most appropriate choice for classifying patients with PDAC into three clusters, namely HIF-1 clusters A, B, and C (**Figures 1A, B**). PCA demonstrated that HIF-1 related genes worked well with significant clustering efficacy (**Figure 1C**). The KM survival curve for OS showed that HIF-1 cluster C had the best survival, and HIF-1 cluster B had shorter medium OS than HIF-1 cluster A, though survival differences between cluster A and cluster B was not statistically significant (**Figure 1D**). A heatmap was constructed to visualize the distribution of these 16 HIF-1 related genes, AJCC stage, and the histologic grade among the three HIF-1 clusters (**Figure 1E**). Furthermore, we found that the HIF-1 cluster was not significantly associated with AJCC stage but significantly associated with histologic grade (**Figures 1F, G**), in which HIF-1 cluster B was significantly correlated with advanced histologic grade and HIF-1 cluster C was correlated with low histologic grade. These results indicate that different HIF-1 clusters are associated with different clinical outcomes.

### Development and Validation of the HIF-1 Score System

**Figure 2:**
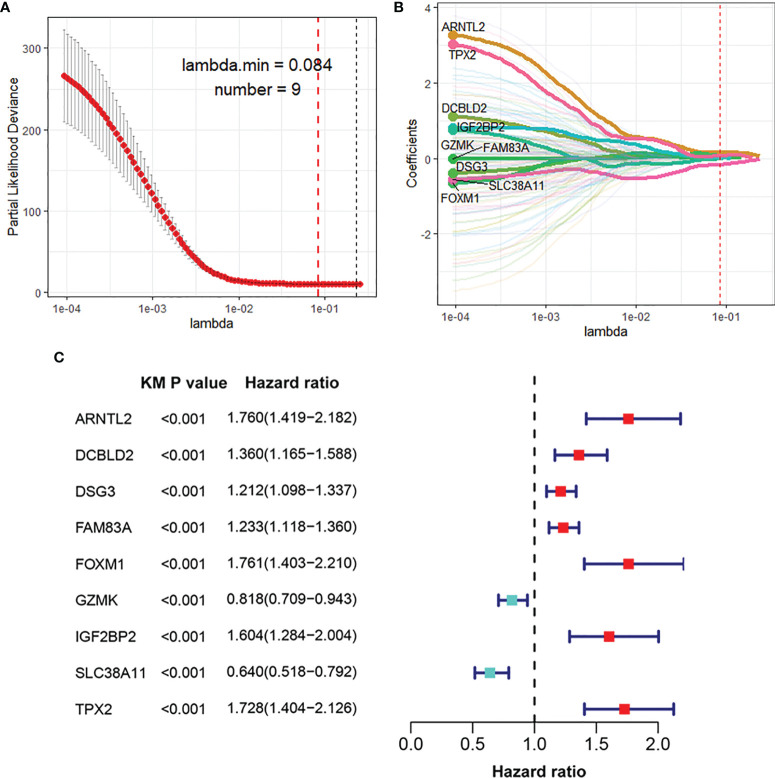
Identification of nine critical prognosis-associated ODEGs for PDAC. **(A, B)** LASSO regression analysis identified nine critical prognosis-associated ODEGs for PDAC. **(C)** Forest plots to show the results of KM survival analysis of the nine critical prognosis ODEGs. ODEGs, overlapping differentially expressed genes; PDAC, pancreatic ductal adenocarcinoma; KM, Kaplan-Meier.

**Figure 3:**
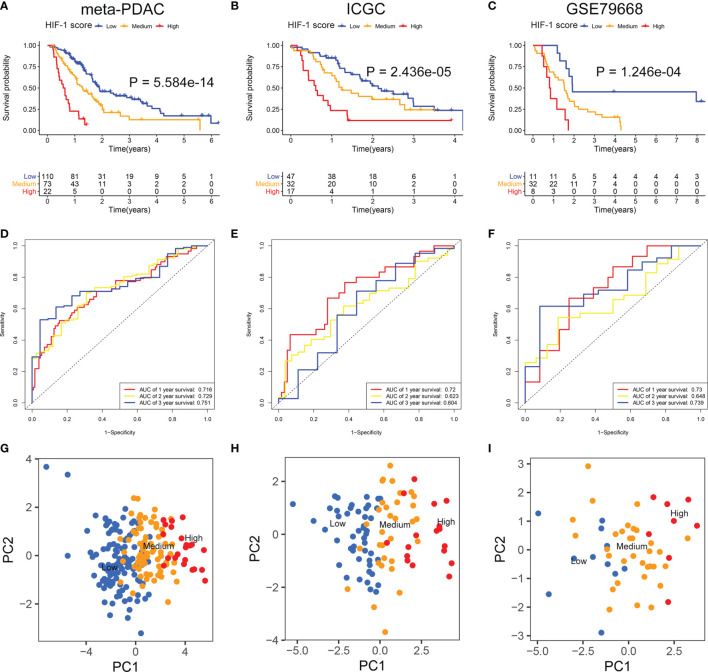
Development and validation of the HIF-1 score system. **(A–C)** KM survival curves for OS of patients with PDAC according to the HIF-1 score groups in the meta-PDAC cohort, ICGC PDAC cohort, and GSE79668 cohort. **(D–F)** ROC curve analysis of the HIF-1 score system for 1-, 2-, and 3-year OS prediction in the meta-PDAC cohort, ICGC PDAC cohort, and GSE79668 cohort. **(G–I)** PCA to confirm the cluster efficacy of the HIF-1 score system in the meta-PDAC cohort, ICGC PDAC cohort, and GSE79668 cohort. KM, Kaplan-Meier; OS, overall survival; PDAC, pancreatic ductal adenocarcinoma; PCA, principal component analysis; ROC, the receiver operating characteristic curve.

The DEGs between different HIF-1 clusters were visualized using volcano plots (**Figures S2A–C**). There were 249 ODEGs among the three HIF-1 clusters (**Figure S2D**). Univariable Cox regression analysis demonstrated that 130 ODEGs were significantly associated with OS of patients with PDAC (P < 0.05) (**Table S2**). Moreover, LASSO regression analysis identified nine critical prognosis-associated ODEGs, and the KM survival analysis is shown in the forest plots (**Figure 2**). Subsequently, based on these nine critical prognosis-associated ODEGs, we constructed an HIF-1 score system using multivariable Cox regression analysis in the meta-PDAC cohort (training cohort). The HIF-1 score was calculated by multiplying the expression of these nine critical prognosis-associated ODEGs by the corresponding multivariable Cox regression coefficients: HIF-1 score = (0.159789 × the expression value of ARNTL2) + (-0.005245 × the expression value of TPX2) + (0.105759 × the expression value of DCBLD2) + (0.045574 × the expression value of IGF2BP2) + (-0.088258 × the expression value of GZMK) + (-0.001925 × the expression value of FAM83A) + (-0.245471 × the expression value of SLC38A11) + (0.214995 × the expression value of FOXM1) + (0.103582 × the expression value of DSG3). Then, we classified patients in the meta-PDAC cohort into high-, medium-, and low-HIF-1 score groups according to the optimal cutoff point obtained from X-tile 3.6.1 software (low HIF-1 score, < 1.006; medium HIF-1 score, 1.006 ≥ & < 2.349; high HIF-1 score, ≥ 2.349). The KM survival curves for OS showed significant differences among these three HIF-1 score subgroups, in which the low-HIF-1 score group had the best survival and the high-HIF-1 score group had the worst survival (**Figure 3A**). For validation, HIF-1 scores were also calculated for 96 patients with PDAC in the ICGC PDAC cohort (validation cohort 1) and 51 patients with PDAC in the GSE79668 cohort (validation cohort 2). Similar results of KM survival analysis were also observed in the validation cohorts (**Figures 3B, C**).

The C-index of the HIF-1 score system for OS prediction in the meta-PDAC cohort were 0.67 (95%CI, 0.62–0.72). For validation cohorts, the HIF-1 score system also exhibited a high accuracy of OS prediction, with a C-index of 0.63 (95%CI, 0.56–0.70) in the ICGC PDAC cohort and a C-index of 0.65 (95%CI, 0.57–0.73) in the GSE79668 cohort. In addition, for the meta-PDAC cohort, the AUC values of the HIF-1 score system for predicting 1-, 2-, and 3-year OS were 0.716, 0.729, and 0.751, respectively (**Figure 3D**). Consistently, the AUC of the HIF-1 score for 1-, 2-, and 3-year OS were 0.720, 0.623, and 0.604 in the ICGC PDAC cohort, and 0.730, 0.648, and 0.739 in the GSE79668 cohort, respectively (**Figures 3E, F**). PCA confirmed the cluster efficacy of the HIF-1 score system (**Figures 3G–I**). These results suggest an optimal discrimination of prognostic prediction using the HIF-1 score system for PDAC.

### Association Between HIF-1 Score and Clinicopathological Characteristics in PDAC

**Figure 4:**
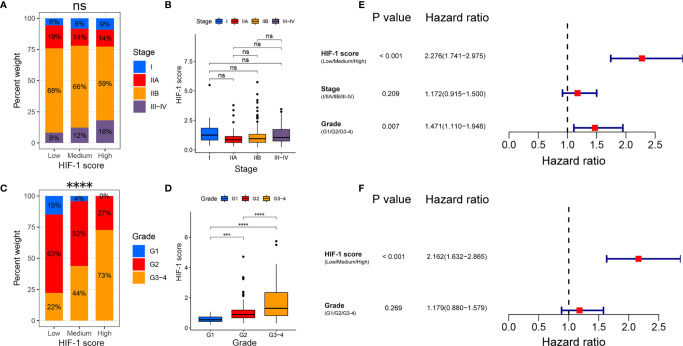
Association between the HIF-1 score and clinicopathological characteristics in PDAC. **(A, B)** No significant association between HIF-1 score and AJCC stage were found in PDAC. **(C, D)** Significant positive correlation between HIF-1 score and histologic grade were observed in PDAC. **(E)** Univariate Cox regression analysis demonstrated that HIF-1 score system and histologic grade were prognostic factor for OS of patients with PDAC. **(F)** Multivariate Cox regression analysis demonstrated that the HIF-1 score system was an independent unfavorable prognostic factor for OS of patients with PDAC. PDAC, pancreatic ductal adenocarcinoma; OS, overall survival. ns > 0.05; ***P value < 0.001; ****P value < 0.0001.

Chi-square analysis indicated that the HIF-1 score system was not significantly correlated with AJCC stage in patients with PDAC, and no significant difference in HIF-1 score was found among different AJCC stages (**Figures 4A, B**). However, the HIF-1 score system was significantly associated with advanced histologic grade, and PDAC patients with higher histologic grade had higher HIF-1 scores than those with lower histologic grades (**Figures 4C, D**). In addition, univariate and multivariate Cox regression analyses demonstrated that the HIF-1 score system was an independent prognostic factor for OS in patients with PDAC (**Figures 4E, F**).

### Association Between HIF-1 Score and Genomic Alteration

**Figure 5:**
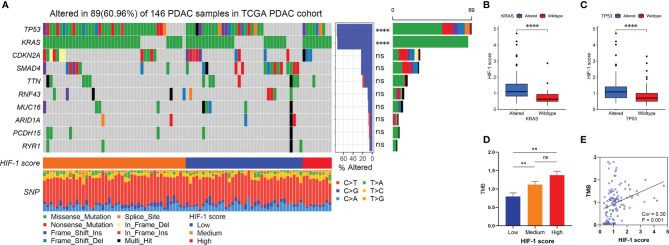
Association between HIF-1 score and genomic alteration in PDAC. **(A)** The waterfall plot shows the top 10 most commonly mutated genes in patients with PDAC. HIF-1 score was significantly correlated with KRAS and TP53 mutation. **(B)** Higher HIF-1 score was found in KRAS altered PDAC. **(C)** Higher HIF-1 score was found in TP53 altered PDAC. **(D)** TMB in low HIF-1 score group PDAC was much lower than those in medium and high HIF-1 score group PDAC. **(E)** HIF-1 score was positively associated with TMB in PDAC. PDAC, pancreatic ductal adenocarcinoma; TMB, tumor mutation burden. ns > 0.05; **P value < 0.01; ****P value < 0.0001.

In line with published studies, we verified that mutations in *KRAS*, *TP53*, *CDKN2A*, and *SMAD4* are four of the most frequent genetic alterations in PDAC (**Figure 5A**). The most common nucleotide change was the C > T transversion (**Figure 5A**). Furthermore, our study revealed that *KRAS* and *TP53* mutation status were significantly correlated with the HIF-1 score (**Figure 5A**). Patients with *KRAS* and *TP53* alterations had significantly higher HIF-1 scores than those with wild type *KRAS* and *TP53* (**Figures 5B, C**). In addition, patients with higher HIF-1 scores had a much higher TMB than those with lower HIF-1 scores (**Figures 5D, E**).

### Analysis of Biological Pathways Among Different HIF-1 Score Groups

**Figure 6:**
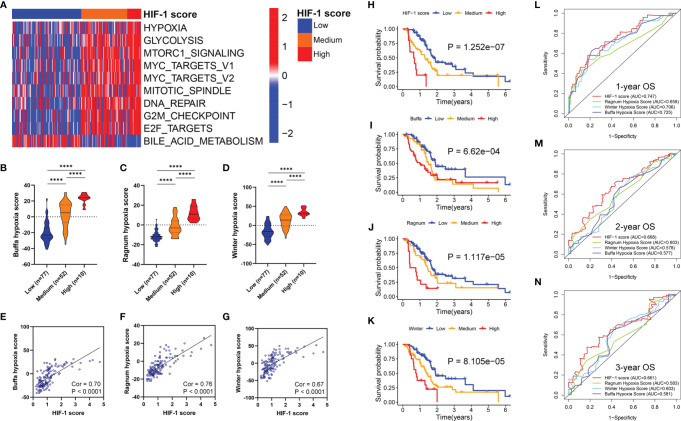
Association between HIF-1 score and hypoxia status and oncologic biological processes in PDAC. **(A)** Heatmap to show the ten critical oncologic biological pathways intersected among the three HIF-1 clutters. **(B–D)** Stepwise scores from HIF-1 score low to high group based on the Buffa hypoxia, Ragnum hypoxia score, and Winter hypoxia score system. **(E–G)** HIF-1 score was significantly correlated with Buffa hypoxia, Ragnum hypoxia score, and Winter hypoxia score system. **(H–K)** KM survival curves for OS of patients with PDAC according to the HIF-1 score system, Buffa hypoxia, Ragnum hypoxia score, and Winter hypoxia score system in TCGA PDAC cohort. **(L–N)** ROC curve analysis for 1-, 2-, and 3-year OS prediction based on the HIF-1 score system, Buffa hypoxia, Ragnum hypoxia score, and Winter hypoxia score system in TCGA PDAC cohort. PDAC, pancreatic ductal adenocarcinoma; KM, Kaplan-Meier; OS, overall survival; TCGA, the Cancer Genome Atlas; ROC, the receiver operating characteristic curve. ****P value < 0.0001.

The top 20 differential oncologic biological pathways and metabolic processes between different HIF-1 score groups were presented using heatmaps (**Figure S3**). Ten critical oncologic biological pathways were found intersected among these three HIF-1 clutters (**Figure 6A**). The high HIF-1 score group had the highest enrichment scores for hypoxia, glycolysis, mTORC1 signaling, MYC signaling (MYC target V1 and MYC target V2), mitotic spindle, DNA repair, G2M targets, and E2F targets, while the low-HIF-1 score group showed the lowest enrichment. By analyzing the metabolic process, we found that the high HIF-1 score group had significant enrichment of nucleobase metabolic processes (**Figure S3E, F**). However, cGMP metabolic process, on the contrary, was significantly downregulated in the high HIF-1 score group but upregulated in the low-HIF-1 score group (**Figure S3E**).

### Comparison With Our HIF-1 Score System With Other Hypoxia Score Systems

Previous studies have reported the hypoxia score system in other cancer types based on transcriptional data. In this study, we compared the predictive ability of our HIF-1 score system with these published hypoxia score systems in pancreatic cancer. First, we observed stepwise scores from HIF-1 low to high score groups calculated by the Buffa hypoxia, Ragnum hypoxia, and Winter hypoxia score systems (**Figures 6B–D**). These results validated our HIF-1 score system reflecting hypoxia status in pancreatic cancer. Furthermore, we found that HIF-1 score was significantly correlated with these three hypoxia score systems (Cor = 0.70, P < 0.0001 for Buffa hypoxia score; Cor = 0.76, P < 0.0001 for Ragnum hypoxia score; Cor = 0.67, P < 0.0001 for Winter hypoxia score) (**Figures 6E–G**). We then compared the prognostic stratification ability of the HIF-1 score system and these three hypoxia scores through KM survival analysis in TCGA PDAC cohort, which showed that the HIF-1 score system had the best performance in prognostic stratification (**Figures 6H–K**). We also compared the discriminatory ability of the HIF-1 score system and these three hypoxia scores in prognostic prediction through AUC of the ROC curves, which demonstrated that the HIF-1 score system had the best predictive efficacy for 1-, 2-, and 3-year OS of PDAC patients (**Figures 6L–N**). These results suggest that our score system had superior predictive ability for patient prognoses.

### Tumors With High HIF-1 Score Were Associated With Immunosuppressive Phenotype

**Figure 7:**
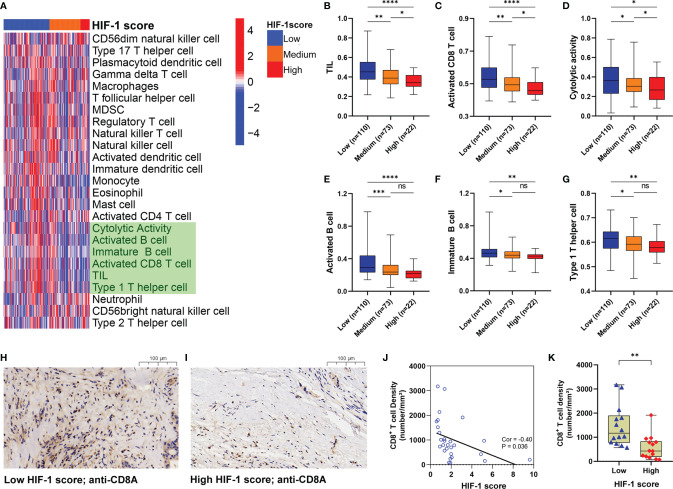
Tumors with high HIF-1 score were associated with immunosuppressive phenotype. **(A)** Heatmap to show the 25 immune-related terms in the three HIF-1 score groups. **(B–G)** Differences of TIL, activated CD8^+^ T cell, cytolytic activity, activated B cell, immature B cell, and Type 1 T helper cell among the three HIF-1 score groups. **(H)** Representative image of CD8A immunostaining in low-HIF-1 score tumor. **(I)** Representative image of CD8A immunostaining in high-HIF-1 score tumor. **(J)** Negative association between the HIF-1 score and CD8^+^ T cell infiltration. **(K)** Tumors with high HIF-1 scores exhibited decreased CD8^+^ T cell infiltration compared with those with low HIF-1 scores. TIL, tumor-infiltrating lymphocytes. ns > 0.05; *P value < 0.05; **P value < 0.01;***P value < 0.001; ****P value < 0.0001.

The 25 immune-related terms were visualized using a heatmap, including CD56dim natural killer cells, type-17 T-helper cells, plasmacytoid dendritic cells, gamma delta T cells, macrophages, T follicular helper cells, myeloid-derived suppressor cells, regulatory T cells, natural killer T cells, natural killer cells, activated dendritic cells, immature dendritic cells, monocytes, eosinophils, mast cells, activated CD4^+^ T cells, cytolytic activity, activated CD8^+^ T cells, tumor-infiltrating lymphocytes (TILs), type 1 T-helper cells, neutrophils, CD56bright natural killer cells, and type 2 T-helper cells (**Figure 7A**). Furthermore, significant differences in the infiltration and cytolytic activity of TILs were observed among different HIF-1 score groups, especially for activated CD8^+^ T cells, B cells (activated and immature), and type 1 T-helper cells (**Figures 7B–G**). Of note, patients in the high HIF-1 score group exhibited the lowest infiltration and cytolytic activity of CD8+ T cells, while those in the low-HIF-1 score group exhibited the highest (**Figures 7C, D**). To further confirm these findings, we investigated the correlation between the HIF-1 score and the infiltration of CD8^+^ T cell using our own dataset. Based on the results of RT-qPCR, we calculated the HIF-1 score for the 28 PDAC tissues, and divided them into low- and high-HIF-1 score according to the medium cutoff of HIF-1 scores. Representative image of CD8A immunostaining of low- and high-HIF-1 score PDAC samples were shown in **Figures 7H, I**. Negative association between the HIF-1 score and CD8^+^ T cell infiltration was observed (**Figure 7J**). And tumors with high HIF-1 scores exhibited decreased infiltration of CD8^+^ T cells compared with those with low HIF-1 score (**Figure 7K**).

We also found that higher HIF-1 scores were significantly correlated with higher PD-L1 and B7-H3 expression in PDAC, which are important molecules regulating the immunosuppressive phenotype (**Figures S4A, B**). In addition, we assessed the differences in HIF-1 scores between various subtypes defined by Bailey et al., which were squamous, immunogenic, pancreatic progenitor, and aberrantly differentiated endocrine exocrine (ADEX) (36). In their study, the squamous subtype showed significantly increased hypoxia response and limited immune infiltration and the worst survival. Consistently, we found that tumors of the squamous subtype had significantly higher HIF-1 scores than those in the immunogenic subtype (“hot tumor”) (**Figure S4C**), suggesting that the squamous subtype had a highly hypoxic status. These results indicate that the HIF-1 score system might be an indicator of the immune-cell infiltration profile in PDAC. In particular, our results suggest an immunosuppressive status of tumors with high HIF-1 score (“cold tumor”).

## Discussion

PDAC is one of the most lethal malignancies with a heterogeneous molecular profile and various hypoxic TMEs (13, 37–39). Over the past decade, molecular target therapy and immune checkpoint inhibitors have been breakthrough advancements in the treatment of various malignancies, such as non-small cell lung cancer, hepatocellular carcinoma, and melanoma (40–42). However, limited therapeutic efficacy has been observed in PDAC due to the limited infiltration of anti-tumor immune cells in the hypoxic TME (43–45). Indeed, the hypoxic TME of PDAC contributes significantly to treatment failure of chemotherapy, target therapy, and immunotherapy (46–50). Targeting tumor hypoxia and HIF-1 signaling might be a promising approach to improve the therapeutic response of chemotherapies or immunotherapies for patients with PDAC. However, a previous study by J. Board et al. found no survival improvement with the combination treatment of a hypoxia inhibitor (TH-302) with gemcitabine in advanced PDAC (51). The disappointing results might be due to several reasons, including not recruiting appropriate patients because they did not check the hypoxic status of the PDAC before treatment. Therefore, to facilitate clinicians in individualized treatment decisions, it is of great value to develop a prognostic classifier to assess the hypoxic status and the corresponding molecular profile of PDAC.

In the present study, based on the known 16 HIF-1 related genes, we constructed a consensus clustering analysis to classify PDAC patients from the meta-PDAC cohort into three HIF-1 clusters. We then developed an HIF-1 score system integrated with nine prognosis-associated ODEGs among the three HIF-1 clusters, which showed reliable predictive efficacy for OS of PDAC patients through KM survival analysis, C-indexes, and AUC of the ROC curves in the training cohort and validation cohorts. In addition, by comparing our HIF-1 score system with other hypoxia score systems established based on other cancer types, we found that our model had the highest prognostic predictive value in PDAC. Taken together, we developed a specific HIF-1 score system for PDAC, which reflects the hypoxic status of tumors and has satisfactory predictive value for patient prognoses.

Some of the nine genes utilized in the HIF-1 score system play critical roles in pancreatic carcinogenesis. For example, Wand et al. indicated that *ARNTL2* is involved in pancreatic carcinogenesis by regulating the TGF-β signaling pathway (52). *IGF2BP2* overexpression promotes PDAC progression through the PI3K/Akt signaling pathway (53). *TPX2* is a critical target of the KRAS signaling pathway and a potential therapeutic target in PDAC (54, 55). Parameswaran et al. demonstrated that *FAM83A* overexpression promotes tumor progression through the MEK-ERK signaling pathway in PDAC (56). *DCBLD2* and *DSG* were identified as unfavorable prognostic biomarkers in PDAC (57, 58). Notably, it has been reported that *FOXM1* is overexpressed in hypoxic cancer cells, which is mediated by HIF-1 (59). Cui et al. also demonstrated that *FOXM1* impelled the Warburg effect and tumor progression in PDAC through transcriptional modulation of LDHA expression, indicating that *FOXM1* is a HIF-1 target affecting PDAC metabolism and progression (60). It should be pointed out, based on the method of data mining, that all the nine genes of our model should be HIF-1-related genes. However, many of them have not been reported to be directly regulated by HIF-1. Further studies are needed to investigate how HIF regulates or interacts with these genes.

In our study, we classified pancreatic cancer patients into three groups based on HIF-1 score system, which were HIF-1 low, medium, and high score groups. Compared to HIF-1 low and medium score groups, pancreatic cancers with HIF-1 high scores are considered more aggressive and refractory because they are associated with worse survival and more advanced grade. By exploring the molecular profile between tumors in HIF-1 low, medium, and high score groups, we found significant enrichment of the MYC and mTORC pathways in the HIF-1 high score group. MYC has been identified as one of the main drivers of PDAC initiation and metastasis (61). Pre-clinical studies have found that inhibition of c-MYC induces cell cycle arrest and chemosensitivity and impairs hypoxia signaling in PDAC (62–65). The mTORC1 pathway is also an oncogenic signaling pathway involved in the proliferation of tumor cells through the modulation of autophagy and angiogenesis (66). Of note, a previous studies revealed that mTORC1 upregulated the transcription and translation of HIF-1 (67–69). Everolimus, an inhibitor of mTORC1, has been shown to impair tumor progression in gemcitabine-resistant PDAC by diminishing the Warburg effect (70). However, Wolpin et al. demonstrated that daily everolimus administered as a single agent had little clinical efficacy in patients with gemcitabine-resistant PDAC (71). Similarly, the combination of everolimus with cytotoxic therapies (e.g., gemcitabine and cisplatin) also failed to achieve meaningful therapeutic responses in patients with PDAC (72–74). These results suggest that single-target therapy may not be sufficient for the eradication of pancreatic cancer cells, especially for refractory and chemoresistant cancer cells. Therefore, combination target therapy may be a promising treatment. Our findings indicate that MYC and mTORC pathways are critical driver in HIF-1 high score tumors, which provides a preliminary rationale for combination treatment using HIF-1 inhibitor and MYC inhibitor, or using HIF-1 inhibitor and everolimus in these highly hypoxic and aggressive pancreatic cancers in future studies.

In addition to activation of MYC and mTORC signaling, we revealed that pancreatic cancers with HIF-1 high scores were more immunosuppressive by further investigation into the hypoxia-immune profiles. In particular, tumors with high HIF-1 scores were associated with low infiltration of TILs, including active CD8^+^ T cells, active and immature B cells, and type 1 helper T cells. These tumors had high expression of PD-L1 and B7-H3, which are important immune checkpoint proteins. Tumor hypoxia and HIF-1 activation regulate many processes of anti-tumor immunity, leading to impaired immune responses and immune evasion (75–78). For example, HIF-1 decreases the production of IL2 and IFN-γ by CD8+ T cells, thereby diminishing the cytolytic activity (75). Hypoxia-mediated ROS also results in immunosuppressive and even lethal toxicity in CD8^+^ T cells (76). Interestingly, using a genetic animal model, Lee et al. demonstrated accumulation of HIF-1α in early pancreatic neoplasia but HIF-1α deletion accelerates PDAC initiation by increasing B cell infiltration, suggesting the pro-neoplastic effect of B cells (79). However, the role of B cells in anti-tumor immunity is still controversial (80, 81). Therefore, further studies are needed to clarify the role of B cell immunity in human PDAC and its interaction with HIF-1. Notably, hypoxia-mediated HIF-1 increases the expression of PD-L1 in multiple solid tumors through PTEN/PI3K signaling, thereby inducing anergy or apoptosis of T cells (78, 82, 83). In addition, we found that a higher HIF-1 score was also observed in the squamous tumor subtype defined by Bailey et al., which was characterized by enrichment for hypoxia response, metabolic reprogramming, and MYC signaling and associated with poor prognosis and limited immune infiltration (36). Taken together, pancreatic cancers with higher HIF-1 scores have a more immunosuppressive TME. Tumors with low/medium HIF-1 scores tend to be good candidates for immunotherapy, especially single treatment with PD-1/PD-L1 inhibitor. For highly hypoxic and immunogenic cold tumors, strategies to break immune-cell infiltrating barriers by inhibiting HIF-1 or reducing desmoplasia may be beneficial for strengthening the efficacy of immunotherapy.

Several limitations of the current study should be noticed. First, a multi-cent and large cohort should be performed to validate the prognostic prediction ability of the HIF-1 score system. Second, further experiment studies should be conducted to investigate the underlying mechanisms by which the HIF-1 related genes regulate anti-tumor immunity in PDAC.

In summary, our study established a specific HIF-1 score system to discriminate pancreatic cancers with various degrees of hypoxia status and immunosuppressive TMEs, which provides accurate predictive value for patient prognoses. In addition, we present distinctive molecular profiles and critical oncogenic pathways for tumors with low/medium HIF-1 scores and high HIF-1 scores, which provide distinctive strategies for treating these pancreatic cancers individually.

## Data Availability Statement

The original contributions presented in the study are included in the article/**Supplementary Material**. Further inquiries can be directed to the corresponding authors.

## Ethics Statement

The study was reviewed and approved by the Ethics Committee of Guangdong Provincial People’s Hospital. The patients/participants provided their written informed consent to participate in this study.

## Author Contributions

Conceptualization: HZ, BH, YC, and CZ. Methodology: HZ, BC, and SW. Investigation: HZ, SW, ZDZ, and BC. Writing – original draft: HZ and CZ. Writing – review and editing: HZ, SW, ZDZ, ZM, ZL, CL, ZXZ, YG, and SH. Visualization: HZ. Supervision: HZ, SW, BC, SH, BH, and CZ. Funding acquisition: BC, BH, and CZ. All authors contributed to the article and approved the submitted version.

## Funding

This study was supported by National Natural Science Foundation of China (NO.: 82072637, 82072635, 81672475 and 81702783), High-level Hospital Construction Project (DFJH201921), and Fundamental Research Funds for the Central Universities (y2syD2192230).

## Conflict of Interest

The authors declare that the research was conducted in the absence of any commercial or financial relationships that could be construed as a potential conflict of interest.

## Publisher’s Note

All claims expressed in this article are solely those of the authors and do not necessarily represent those of their affiliated organizations, or those of the publisher, the editors and the reviewers. Any product that may be evaluated in this article, or claim that may be made by its manufacturer, is not guaranteed or endorsed by the publisher.

## Supplementary Material

Supplementary Figure 1 **(A)** Differential expression analysis of the 16 HIF-1 related genes between non-tumor and tumor samples. **(B)** Forest plots to show the results of KM survival analysis of the 16 HIF-1 related genes. KM, Kaplan-Meier. **P value < 0.01; ***P value < 0.001; ****P value < 0.0001.

Supplementary Figure 2 **(A)** Differential expression analysis between HIF-1 cluster **(A, B)**. **(B)** Differential expression analysis between HIF-1 cluster **(A, C)**. **(C)** Differential expression analysis between HIF-1 cluster **(B, C)**. **(D)** Intersection of differential expressed genes among these three HIF-1 clusters.

Supplementary Figure 3 Heatmaps to show the top 20 differential oncologic biological pathways and metabolic processes between different HIF-1 score groups were respectively presented using heatmaps.

Supplementary Figure 4 **(A)** Association between HIF-1 score and PD-L1 expression in PDAC. **(B)** Association between HIF-1 score and B7-H3 expression in PDAC. **(C)** Patients in patients in squamous subtype had significant higher HIF-1 score than those in immunogenic subtype. PDAC, pancreatic ductal adenocarcinoma. ****P value < 0.0001.
