# Supplementary figures and images for: Prognostic Stratification Based on HIF-1 Signaling for Evaluating Hypoxic Status and Immune Infiltration in Pancreatic Ductal Adenocarcinomas
Source: Front Immunol. 2021 Dec 3;12:790661. doi: 10.3389/fimmu.2021.790661 (PMC8677693; doi:10.3389/fimmu.2021.790661)

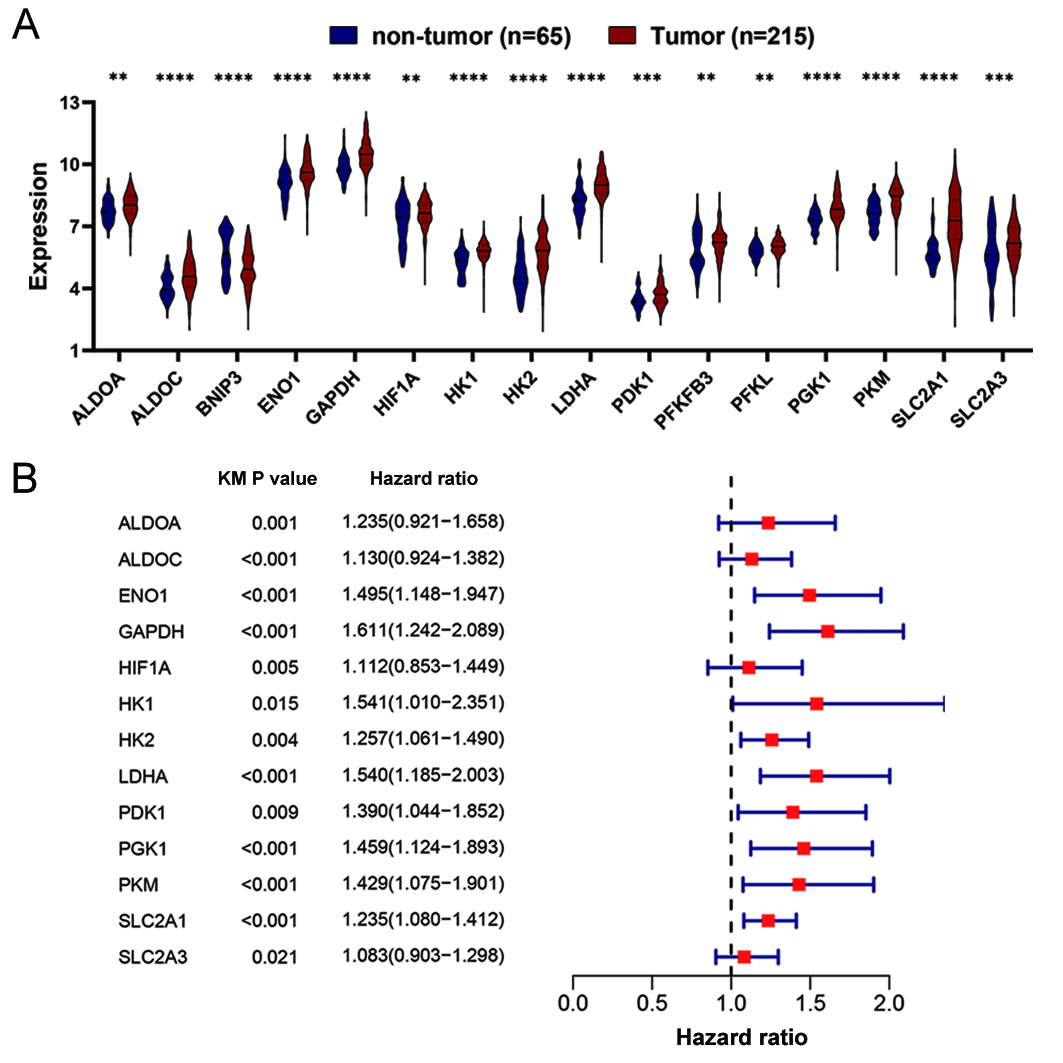

Supplement: Supplementary Figure 1 — (A) Differential expression analysis of the 16 HIF-1 related genes between non-tumor and tumor samples. (B) Forest plots to show the results of KM survival analysis of the 16 HIF-1 related genes. KM, Kaplan-Meier. **P value < 0.01; ***P value < 0.001; ****P value < 0.0001. [file Image_1.tif]

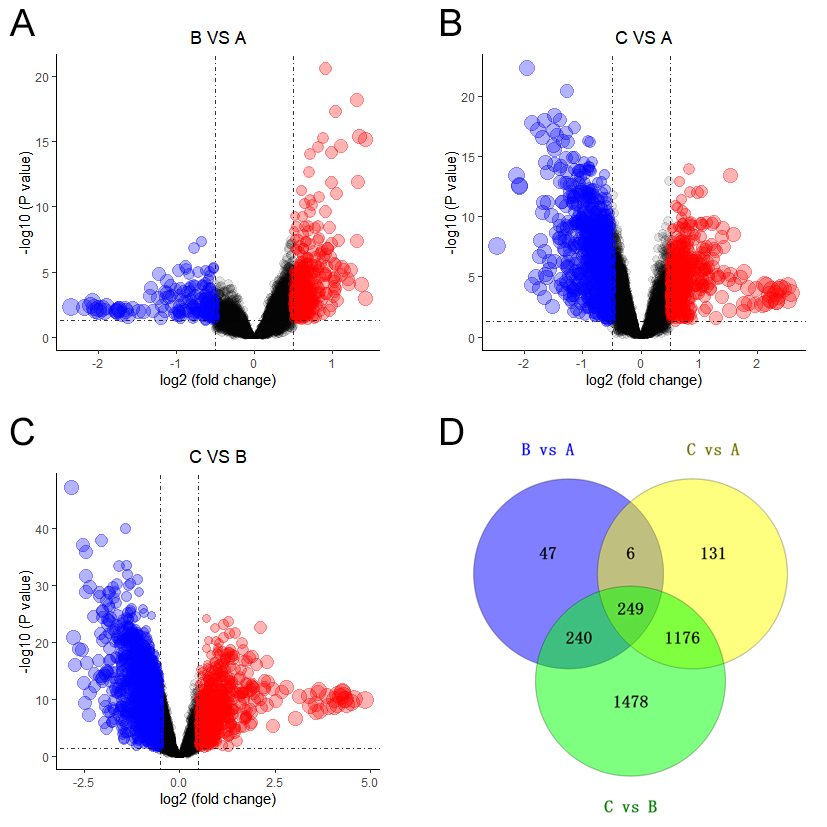

Supplement: Supplementary Figure 2 — (A) Differential expression analysis between HIF-1 cluster (A, B). (B) Differential expression analysis between HIF-1 cluster (A, C). (C) Differential expression analysis between HIF-1 cluster (B, C). (D) Intersection of differential expressed genes among these three HIF-1 clusters. [file Image_2.tif]

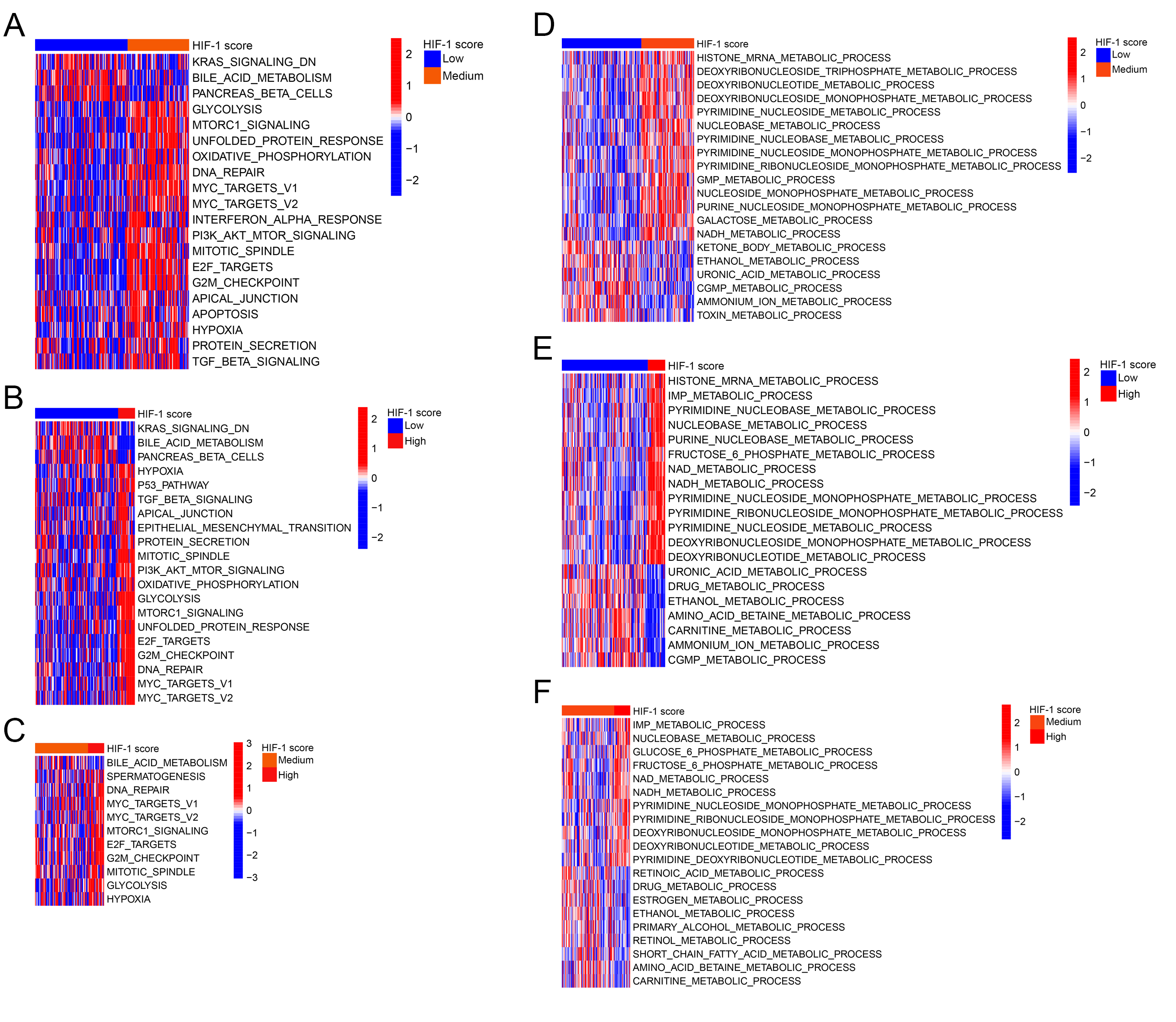

Supplement: Supplementary Figure 3 — Heatmaps to show the top 20 differential oncologic biological pathways and metabolic processes between different HIF-1 score groups were respectively presented using heatmaps. [file Image_3.tif]

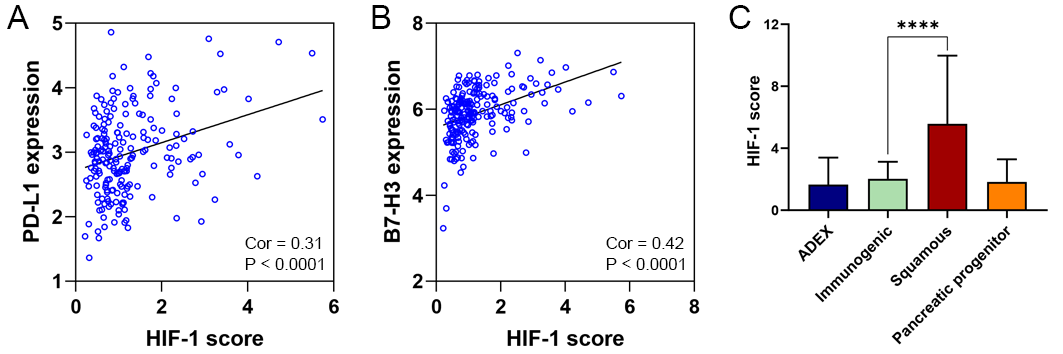

Supplement: Supplementary Figure 4 — (A) Association between HIF-1 score and PD-L1 expression in PDAC. (B) Association between HIF-1 score and B7-H3 expression in PDAC. (C) Patients in patients in squamous subtype had significant higher HIF-1 score than those in immunogenic subtype. PDAC, pancreatic ductal adenocarcinoma. ****P value < 0.0001. [file Image_4.tif]
